# Triage of children in an Emergency Department

**DOI:** 10.1186/1757-7241-21-S2-A49

**Published:** 2013-09-09

**Authors:** Dennis Graversen, Ann-Britt Kiholm Kirkedal

**Affiliations:** 1 Emergency Department, Holbaek Hospital, Denmark; 2 Department of Paediatrics, Holbaek Hospital, Denmark

## Background

To secure the best primary treatment of children a nationwide triage system has been instituted. The aim of this study was to evaluate the primary implementation in an Emergency Department(ED).

## Methods

Through a retrospective evaluation of medical records vital parameters and triage assessments made by an ED nurse were collected. Children younger than 13 years of age with no referral from a doctor were included (from October to December 2012) (n=127).

## Results

A total of 81.9% (n=104) of the children were registered with triage colour indicating the level of urgency. This was done within a mean of 26.4 minutes from time of arrival. Children younger than 1 year old were significantly more likely not to be triaged compared to children older than 1 year old RR 2.78 (95% CI 1.31-5.93) (p=0.0249). Distribution of triage was; green (lowest level) 58.3%, yellow 24.4%, orange 16.5% and red (highest level) 0.9%. In 22.1% of the patients we identified a lower triage level than given with the vital parameters . The greatest risk of being under-triaged was found among children between 3-7 years (39.4%).

## Conclusion

A simple and clear triage system is of great importance in order to get a well functioning triage in an ED. We showed that special attention should be given to infants to secure proper triage and that children from 3-7 years of age is at the highest risk of being under-triaged.

